# Minimal residual disease monitoring by Ig/TCR gene rearrangements predicts post-transplant relapse and survival in adult patients with acute lymphoblastic leukemia

**DOI:** 10.1007/s00277-024-05943-1

**Published:** 2024-08-21

**Authors:** Michael Stephan Bader, Susanne Kubetzko, Beat Werner Schäfer, Christian Arranto, Beatrice Drexler, Jörg Halter, Jakob R. Passweg, Michael Medinger

**Affiliations:** 1 Divisions of Hematology and Internal Medicine, Department of Medicine, University Hospital Basel, Petersgraben 4, Basel, CH-4031 Switzerland; 2 University of Basel, Basel, Switzerland; 3 Division of Oncology, University Children’s Hospital, Zürich, Switzerland

Dear Editor,

Monitoring minimal residual disease (MRD) using immunoglobulin (Ig) and T-cell receptor (TCR) gene rearrangements has become standard practice in current treatment protocols for adult patients with acute lymphoblastic leukemia (ALL). While its prognostic significance following induction and consolidation therapy is widely recognized, its impact in the pre- and post-transplant setting remains less well explored [1–3].

We conducted a retrospective cohort study to assess the impact of MRD detected by Ig/TCR rearrangements on outcomes in adult ALL patients undergoing first allogeneic hematopoietic cell transplantation (HCT) at our center. Out of 136 patients treated between 01/01/2006 and 01/12/2021, 47 had pre- and post-transplant MRD monitored by Ig/TCR rearrangements according to the EuroMRD guidelines (supplementary material) [4, 5].

With a median follow-up of 28.7 months, patients with undetectable MRD before allogeneic HCT had a 5-year overall survival (OS) of 89% and progression-free survival (PFS) of 76%, compared to 68% and 52% in MRD-positive patients (OS, *p* = 0.025; PFS, *p* = 0.044) (supplementary material). Similar outcomes were observed in the *BCR::ABL1*-negative subgroup, comprising 85% of all patients (*p* = 0.048 for both OS and PFS, Fig. 1).

MRD positivity at the 3-month follow-up was strongly linked to disease relapse. In *BCR::ABL1*-negative individuals, the cumulative incidence of relapse at 5 years approached 75% with detectable MRD, as opposed to 15% in patients who remained MRD-negative (*p* = 0.003) (supplementary material). Most interestingly, detection of MRD at three months was associated with a significant decline in OS even when the extent of MRD was below the quantifiable range (*p* = 0.024).

Comparison between flow cytometry and Ig/TCR rearrangements for MRD assessment revealed a notable 18% discordance. These discrepancies mainly occurred when Ig/TCR rearrangements identified MRD without a corresponding blast population in flow cytometry. Remarkably, patients with discordant results three months post-allogeneic HCT had diminished OS compared to those with negative assessments by both methodologies (*p* < 0.001).

We observed a significant difference in the frequency of achieving MRD negativity before transplantation depending on the affected cell line, with B-ALL patients exhibiting a higher rate of molecular remission than patients with T-ALL (*p* = 0.009). This may be attributed to the improved therapeutic options available for relapsed B-ALL, particularly with the advent of bispecific antibodies targeting CD19.

Remarkably, patients harboring a *BCR::ABL1* fusion did not show a diminished probability of attaining MRD negativity based on Ig/TCR rearrangements prior to allogeneic HCT. This observation aligns with findings from the recently published GRAAPH-2014 study on the prognostic role of MRD in Philadelphia chromosome-positive ALL, which highlighted the limited significance of *BCR::ABL1* MRD due to the presence of residual *BCR::ABL1*-positive non-ALL cells in a substantial subset of patients [6].

In conclusion, despite our study’s limitations, our center’s experience supports that Ig/TCR rearrangements for MRD detection before and after allogeneic HCT reliably assess relapse and overall survival in adult ALL patients. Incorporating this method into ALL management holds significant potential to enhance risk stratification and therapeutic decision-making, ultimately contributing to improved clinical outcomes and patient care.

**Fig. 1:**
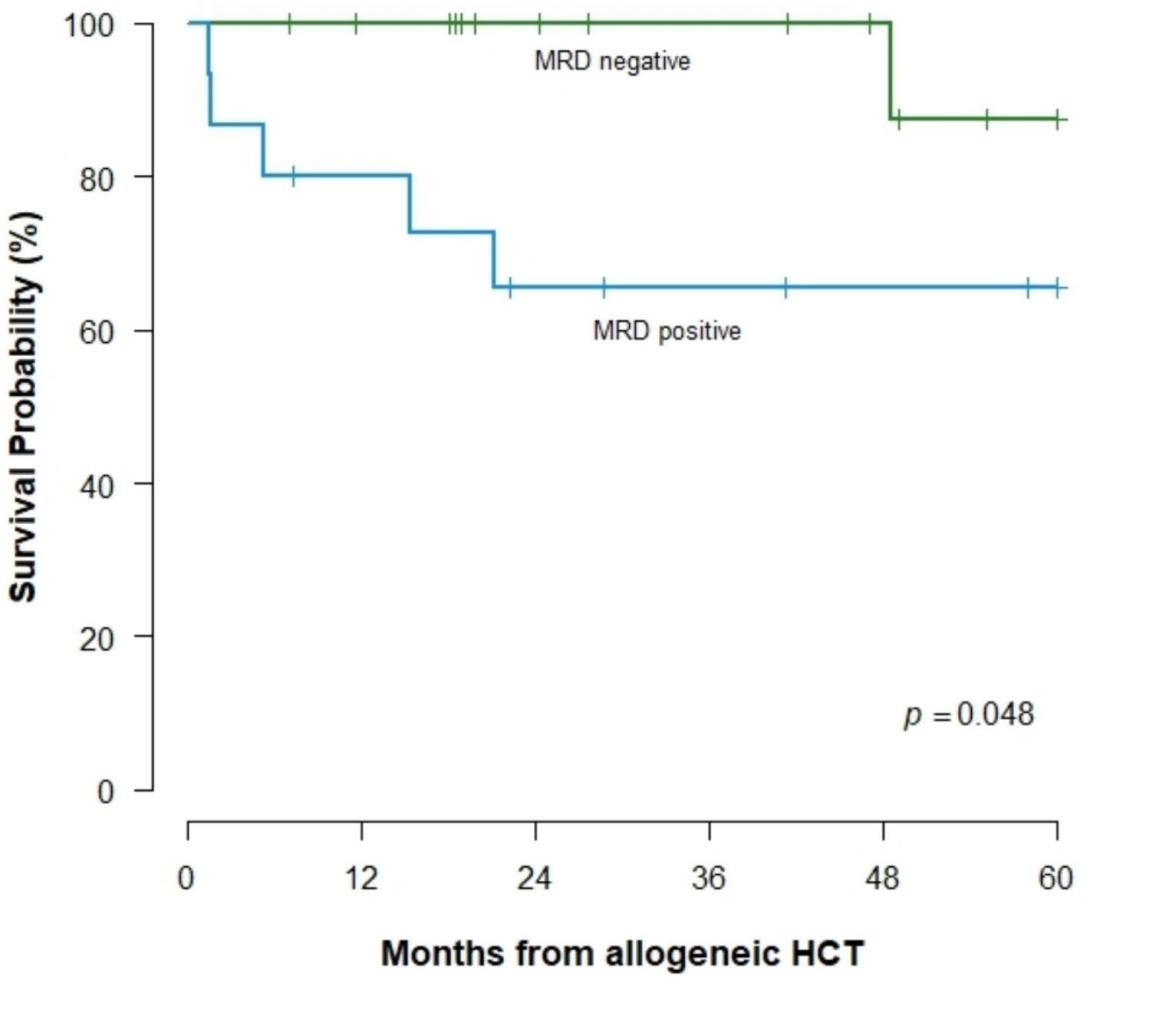
Overall survival of *BCR::ABL1*-negative ALL patients, stratified by MRD using Ig/TCR rearrangements prior to allogeneic HCT

## Electronic supplementary material

Below is the link to the electronic supplementary material.

Supplementary Material 1

## Data Availability

No datasets were generated or analysed during the current study.
