# Supplementary material for: Minimal residual disease monitoring by Ig/TCR gene rearrangements predicts post-transplant relapse and survival in adult patients with acute lymphoblastic leukemia
Source: Ann Hematol. 2024 Aug 21;103(11):4831–3. doi: 10.1007/s00277-024-05943-1 (PMC11534960; doi:10.1007/s00277-024-05943-1)
Supplement: Supplementary file 1 — Supplementary Material 1 [file 277_2024_5943_MOESM1_ESM.docx]

**Supplementary material**

| Variable | All patients  (n = 47) | MRD negative  (n = 26) | MRD positive  (n = 21) | *p* value |
| --- | --- | --- | --- | --- |
| Follow-up from  transplantation |  |  |  |  |
| Median (range) – months | 28.7 (0.9 – 138.3) | 38.5 (2.6 – 120.2) | 21.1 (0.9 – 138.3) | 0.24 |
| Age at transplantation |  |  |  |  |
| Median (range) – years  ≥ 40 years – no. (%) | 40 (18 – 68)  24 (51) | 43 (19 – 68)  17 (65) | 34 (18 – 64)  7 (33) | 0.17 |
| Age at diagnosis |  |  |  |  |
| Median (range) – years  ≥ 40 years – no. (%) | 39 (16 – 68)  21 (45) | 41.5 (16 – 68)  14 (54) | 33 (16 – 63)  7 (33) | 0.26 |
| Sex – no. (%) |  |  |  | 1.00 |
| Male  Female | 29 (62)  18 (38) | 16 (62)  10 (38) | 13 (62)  8 (38) |  |
| Lineage – no. (%) |  |  |  | **< 0.01** |
| B cell lineage  T cell lineage | 34 (72)  13 (28) | 23 (88)  3 (12) | 11 (52)  10 (48) |  |
| *BCR::ABL1* fusion – no. (%) |  |  |  | 0.11 |
| Yes  No | 7 (15)  40 (85) | 6 (23)  20 (77) | 1 (5)  20 (95) |  |
| Karyotype – no. (%) |  |  |  | 0.76 |
| Normal  Abnormal | 18 (38)  29 (62) | 9 (35)  17 (65) | 9 (43)  12 (57) |  |
| Stem cell source – no. (%) |  |  |  | 0.68 |
| Peripheral blood  Bone marrow | 41 (87)  6 (13) | 22 (85)  4 (15) | 19 (90)  2 (10) |  |
| HLA match – no. (%) |  |  |  | 0.52 |
| Identical sibling  Matched unrelated  Haploidentical relative | 21 (45)  19 (40)  7 (15) | 10 (38)  11 (42)  5 (19) | 11 (52)  8 (38)  2 (10) |  |
| Conditioning – no. (%) |  |  |  | 0.68 |
| Myeloablative  Reduced intensity | 41 (87)  6 (13) | 22 (85)  4 (15) | 19 (90)  2 (10) |  |
| Total body irradiation  – no. (%) |  |  |  |  |
| Yes  ≥ 8 Gy  < 8 Gy  No | 40 (85)  35 (88)  5 (12)  7 (15) | 21 (81)  17 (81)  4 (19)  5 (19) | 19 (90)  18 (95)  1 (5)  2 (10) | 0.44  0.35 |
| GvHD prophylaxis – no. (%) |  |  |  | **0.04** |
| Non-ATG-based  ATG-based | 21 (45)  26 (55) | 8 (31)  18 (69) | 13 (62)  8 (38) |  |
| Karnofsky performance score |  |  |  |  |
| Median (range) – %  ≥ 80 – no. (%) | 90 (40 – 100)  35 (74) | 90 (60 – 100)  19 (73) | 90 (40 – 100)  16 (76) | 0.66 |
| Disease status at allogeneic HCT – no. (%) |  |  |  |  |
| Complete remission  1^st^ CR  2^nd^ CR  Other  Advanced disease | 42 (89)  24 (57)  17 (40)  1 (2)  5 (11) | 26 (100)  14 (54)  12 (46)  0 (0)  0 (0) | 16 (76)  10 (62)  5 (31)  1 (6)  5 (24) | **0.01** |
| ABO blood group barrier – no. (%) |  |  |  | 0.23 |
| Major  Minor  Bidirectional  None | 12 (26)  9 (19)  2 (4)  24 (51) | 5 (19)  6 (23)  0 (0)  15 (58) | 7 (33)  3 (14)  2 (10)  9 (43) |  |
| CMV constellation  (donor/recipient) – no. (%) |  |  |  | 0.76 |
| Negative / Negative  Positive / Positive  Negative / Positive  Positive / Negative | 7 (15)  23 (49)  8 (17)  9 (19) | 4 (15)  11 (42)  5 (19)  6 (23) | 3 (14)  12 (57)  3 (14)  3 (14) |  |

**Table 1:** Demographic and clinical characteristics of the patients and univariable analysis of association with Ig/TCR-based MRD status prior to allogeneic HCT.


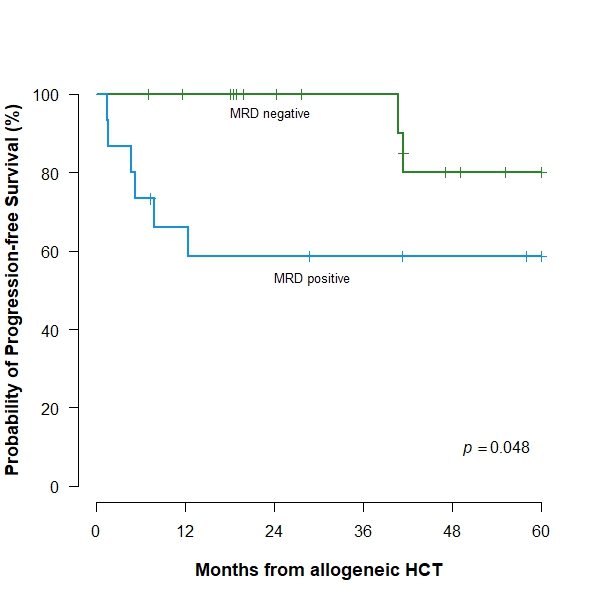


**Figure S1:** Progression-free survival of *BCR::ABL1*-negative ALL patients, stratified by measurable residual disease using Ig/TCR rearrangements prior to allogeneic hematopoietic cell transplantation.


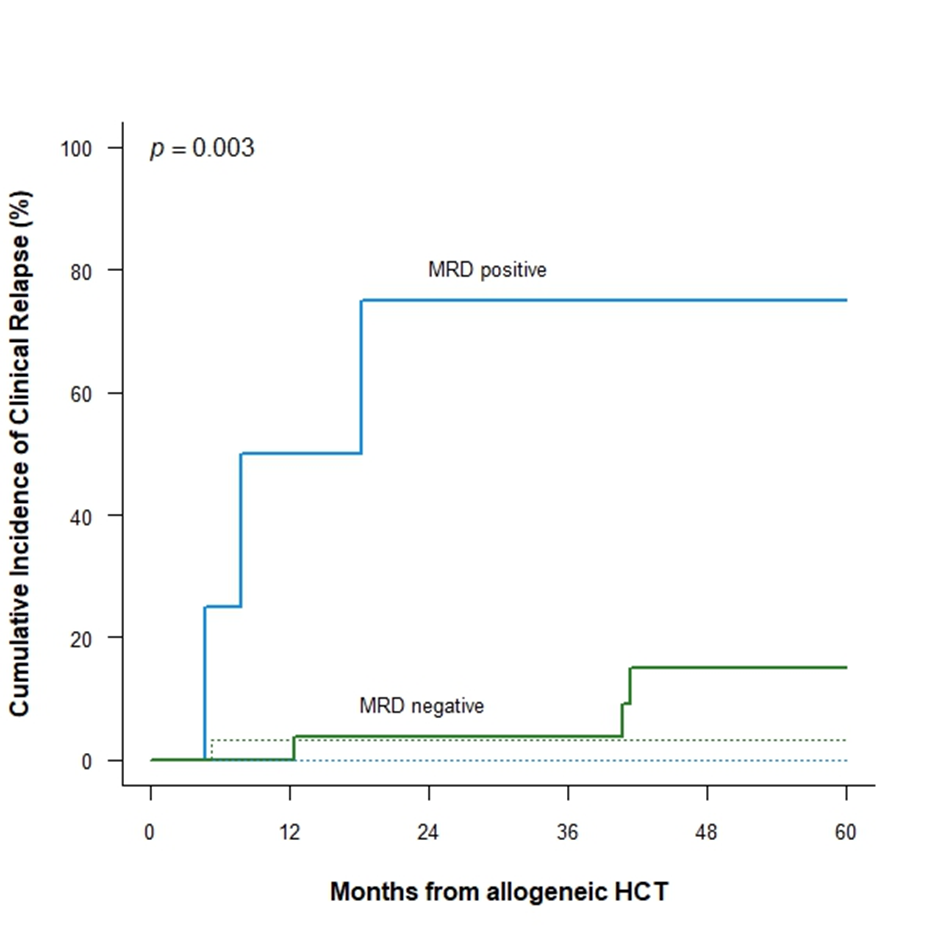


**Figure S2:** Cumulative incidence of clinical relapse in the *BCR::ABL1*-negative cohort depending on MRD detection three months after allogeneic HCT. Dashed curves depict the cumulative incidence of competitive events (death without disease recurrence).


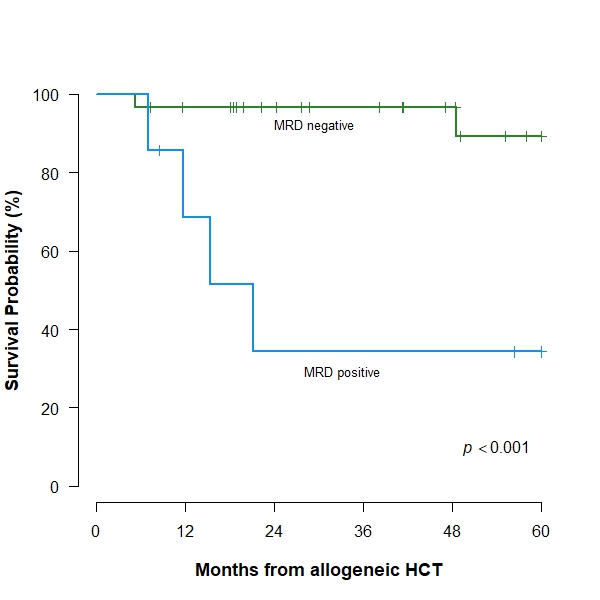
(**a**)


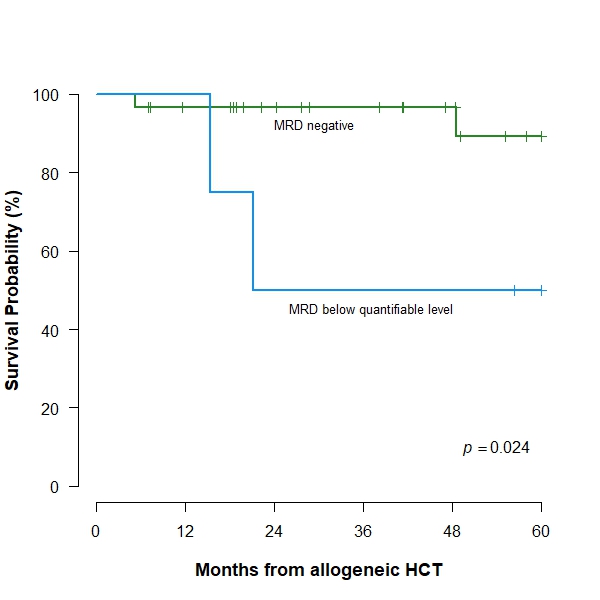
(**b**)

**Figure S3:** Overall survival in the *BCR::ABL1*-negative cohort based on MRD detection three months after allogeneic HCT. **(a)** Comparison between MRD-positive and MRD-negative patients; **(b)** Comparison between patients with MRD below the quantifiable range and MRD-negative individuals.


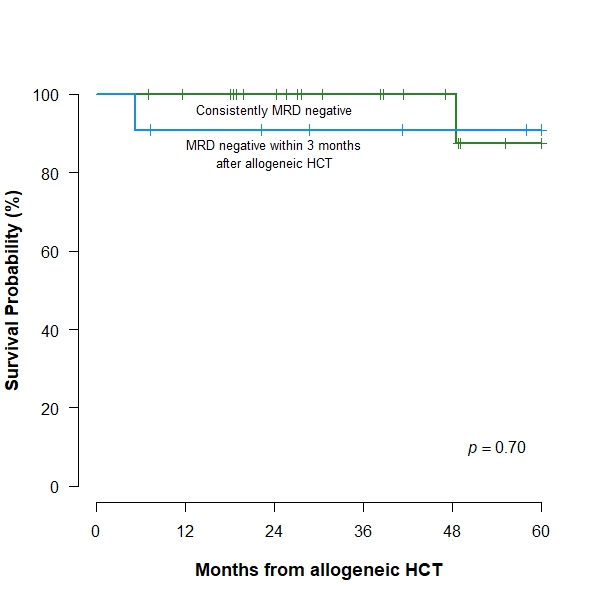


**Figure S4:** Comparison of overall survival between patients achieving MRD negativity within three months after allogeneic HCT and individuals with consistent MRD negativity.


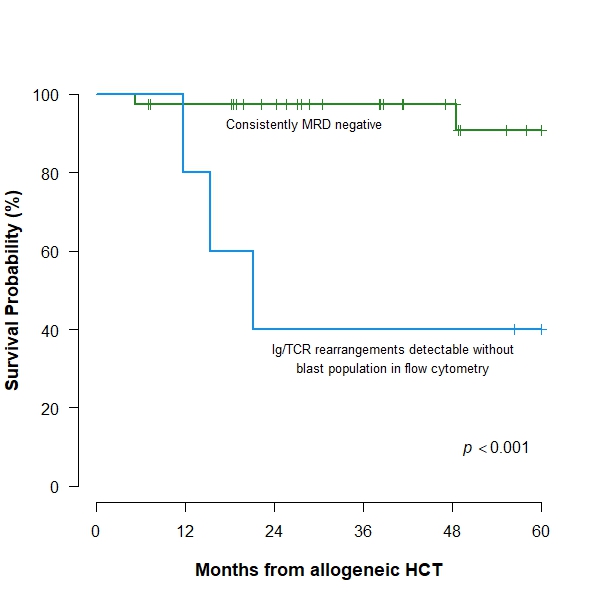


**Figure S5:** Overall survival, stratified by negative MRD detection three months after allogeneic HCT with both multicolor flow cytometry and analysis of Ig/TCR rearrangements or detection of Ig/TCR rearrangements only.
